# Chromosome 17q12 microdeletions but not intragenic *HNF1B* mutations link developmental kidney disease and psychiatric disorder

**DOI:** 10.1016/j.kint.2016.03.027

**Published:** 2016-07

**Authors:** Rhian L. Clissold, Charles Shaw-Smith, Peter Turnpenny, Benjamin Bunce, Detlef Bockenhauer, Larissa Kerecuk, Simon Waller, Pamela Bowman, Tamsin Ford, Sian Ellard, Andrew T. Hattersley, Coralie Bingham

**Affiliations:** 1 University of Exeter Medical School, Exeter, UK; 2 National Institute for Health Research Exeter Clinical Research Facility, Royal Devon and Exeter National Health Service Foundation Trust, Exeter, UK; 3 Clinical Genetics Department, Royal Devon and Exeter National Health Service Foundation Trust, Exeter, UK; 4 Department of Molecular Genetics, Royal Devon and Exeter National Health Service Foundation Trust, Exeter, UK; 5 Department of Nephrology, Great Ormond Street Hospital for Children National Health Service Foundation Trust, London, UK; 6 University College of London Centre for Nephrology, London, UK; 7 Department of Nephrology, Birmingham Children’s Hospital, Birmingham, UK; 8 Department of Nephrology, Evelina London Children's Hospital, St. Thomas' Hospital, London, UK; 9 Macleod Diabetes and Endocrine Centre, Royal Devon and Exeter National Health Service Foundation Trust, Exeter, UK; 10 Exeter Kidney Unit, Royal Devon and Exeter National Health Service Foundation Trust, Exeter, UK

**Keywords:** 17q12 deletion, cystic kidneys, developmental kidney disease, *HNF1B*, neurodevelopmental disorders

## Abstract

Heterozygous mutations of the *HNF1B* gene are the commonest known monogenic cause of developmental kidney disease. Half of patients have a deletion (approximately 1.3 Mb) of chromosome 17q12, encompassing *HNF1B* plus 14 additional genes. This 17q12 deletion has been linked with an increased risk of neurodevelopmental disorders, such as autism. Here we compared the neurodevelopmental phenotype of 38 patients with HNF1B-associated renal disease due to an intragenic mutation in 18 patients or due to 17q12 deletion in 20 patients to determine whether haploinsufficiency of *HNF1B* is responsible for the neurodevelopmental phenotype. Significantly, brief behavioral screening in children with the deletion showed high levels of psychopathology and its impact. Eight individuals (40%) with a deletion had a clinical diagnosis of a neurodevelopmental disorder compared to none with an intragenic mutation. The 17q12 deletions were also associated with more autistic traits. Two independent clinical geneticists were able to predict the presence of a deletion with a sensitivity of 83% and specificity of 79% when assessing facial dysmorphic features as a whole. Thus, the 17q12 deletions but not *HNF1B* intragenic mutations are associated with neurodevelopmental disorders. Hence, the *HNF1B* gene is not involved in the neurodevelopmental phenotype of these patients. Nephrologists need to be aware of this association to ensure appropriate referral to psychiatric services.

Heterozygous mutations in the gene encoding the transcription factor hepatocyte nuclear factor 1β (HNF1B) are the commonest known monogenic cause of developmental kidney disease.^1^, ^2^, ^3^ The phenotype of HNF1B-associated renal disease is very variable despite this single genetic etiology. Abnormalities are often detected on prenatal ultrasonography, where bilateral hyperechogenic kidneys with normal or slightly increased size are commonly found.^4^ Cystic disease, including cystic dysplasia, is usually seen in both pediatric and adult populations but other developmental kidney disease has been reported, including single kidneys, hypoplasia, horseshoe kidneys, duplex kidneys, collecting system abnormalities, bilateral hydronephrosis and hyperuricemic nephropathy.^1^, ^5^, ^6^, ^7^, ^8^, ^9^, ^10^, ^11^ Electrolyte abnormalities, including hypomagnesemia and hyperuricemia, are common.^10^, ^11^ HNF1B-associated disease is a multisystem disorder; extrarenal phenotypic features include early onset diabetes mellitus, pancreatic hypoplasia, genital tract malformations, and abnormal liver function tests.^12^, ^13^, ^14^, ^15^, ^16^, ^17^ Genetic changes in the *HNF1B* gene comprise either whole-gene deletions (approximately one-half of patients) or intragenic mutations (base substitutions or small insertions/deletions within the *HNF1B* gene).^8^, ^18^ Both may arise spontaneously; 50% of whole-gene deletions are *de novo*.^4^, ^7^, ^19^ This means there is frequently no family history of renal disease or diabetes.

The majority of patients with a whole-gene deletion have an approximate 1.3 Mb deletion at chromosome 17q12, which includes the entire *HNF1B* gene.^20^ These recurrent microdeletions of 17q12 are mediated by flanking segmental duplications via nonallelic homologous recombination.^21^ Unlike most genomic disorders, the 17q12 deletion was not initially thought to be associated with developmental delay or intellectual disability. More recent work has shown that neurodevelopmental disorders, including autism spectrum disorders (ASD), are part of the phenotype in patients referred for testing via clinical genetics rather than renal services.^22^, ^23^, ^24^, ^25^, ^26^ A study by Laffargue *et al.*^20^ suggests that the neuropsychological phenotype is less severe than that previously reported when the 17q12 deletion is identified secondary to renal abnormalities. Comparison of 26 children with *HNF1B* deletions and 13 with point mutations under the care of pediatric nephrologists showed no significant differences in relation to learning abilities and schooling, although the deletion group tended to have lower intelligence quotients (IQs) and more educational difficulties at school than those with a mutation. However, formal neuropsychological evaluation was only carried out in a small subset of the cohort (11 of 39) and several of the children included in the study were too young to evaluate for schooling difficulties and ASD.

The 1.3 Mb deleted region contains 14 genes in addition to *HNF1B* and it is not clear what genetic mechanism gives rise to this neurodevelopmental phenotype. One hypothesis is haploinsufficiency of 1 of these 15 genes. *HNF1B* is involved in hindbrain development in both zebra fish and mice and so is a good candidate to be the etiological gene.^27^, ^28^ There have been rare reports of learning difficulties and epilepsy in 5 patients with *HNF1B* gene mutations, which would support this.^6^, ^12^, ^29^ Another candidate is *LHX1*, which is also expressed in the brain during early development; a mouse model with a targeted mutation of *Lhx1* confirms its role as a key regulator of the vertebrate head organizer.^30^, ^31^ A study investigating new hotspots of copy-number variation associated with ASD has implicated *ACACA* within the 17q12 deletion.^32^ However, no single gene deletions or mutations resulting in haploinsufficiency and neurological disease in humans have been detected in either of these genes to date. An alternative hypothesis would involve more complex interactions between genes within the deleted 17q12 region and other transcription factors giving rise to an increased risk of neurodevelopmental disorders.^20^ In this study, we systematically compared the neurodevelopmental phenotype of patients with either an *HNF1B* intragenic mutation or 17q12 deletion to determine whether haploinsufficiency of the *HNF1B* gene is responsible for this aspect of the phenotype.

## Results

### General characteristics are similar in both *HNF1B* mutation and deletion groups

Thirty-eight individuals participated in the study; 18 (47%) had a known intragenic *HNF1B* mutation and 20 (53%) had a whole-gene deletion. The intragenic mutations included 4 nonsense, 13 insertions/deletions, and 1 missense change (Supplementary Table S1). The presence of the common 1.3 Mb 17q12 deletion was confirmed by dosage analysis of *ZNHIT3* and *HNF1B*, the most 5′ and 3′ of the 15 genes within the interval, by droplet digital polymerase chain reaction (PCR) in the deletion group (data not shown).

Both mutation and deletion groups were similar in terms of general characteristics (Table 1). Median age at study inclusion was similar between the groups, as was sex breakdown. Participants were predominantly of White British origin, reflecting the fact that 61% were recruited from South West England. Levels of deprivation were similar in both mutation and deletion groups as measured using the median Index of Multiple Deprivation 2007 score. Cysts or cystic dysplasia were the renal phenotype seen most commonly in both groups, similar to cohorts with HNF1B-associated kidney disease previously described in the literature.^5^ Renal function was worse in the mutation group with a median estimated glomerular filtration rate (GFR) of 42.6 ml/min/1.73 m^2^ (interquartile range [IQR]: 31, 60) compared with 81.4 (IQR: 56, 91) in the deletion group, *P* = 0.002. Serum magnesium levels were lower in the deletion group, whereas hyperuricemia was more common in the mutation group; both of these findings may be explained by the greater degree of renal impairment seen in the mutation group. Diabetes was present in approximately 40% of patients in both groups. Other extrarenal phenotypes were also similar between mutation and deletion groups.

The 38 patients included in this study represented 45% of those with HNF1B-associated disease who were eligible to take part from the 4 different sites. Supplementary Table S2 compares the general characteristics of participants and nonparticipants. Briefly, the 2 groups were similar in terms of genetic abnormality, age, sex, levels of deprivation, and renal phenotype. The only difference was in ethnicity, with other ethnic groups besides White British being more commonly represented among nonparticipants. However, the data available for nonparticipants was incomplete with 13 of 47 (28%) having no information on ethnicity recorded.

### Brief behavioral screening shows higher levels of psychopathology and impact in children with a deletion

**Figure S1:**
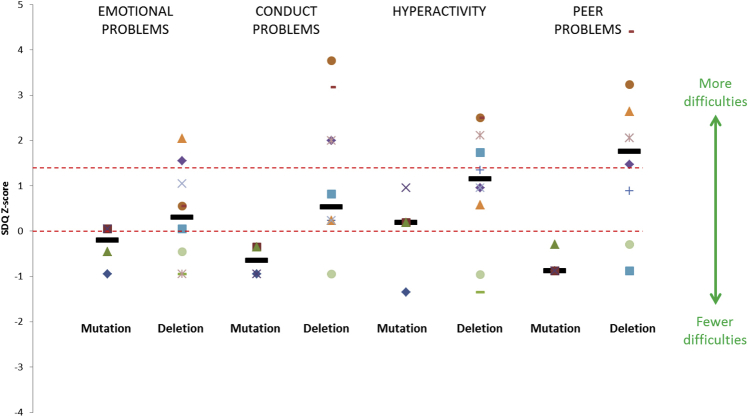
Patient difficulties as shown by subsections of the parent-reported Strengths and Difficulties Questionnaire (SDQ) scores (presented as *Z*-scores) for individuals <18 years with both *HNF1B* gene mutations (*n* = 4) and 17q12 microdeletions (*n* = 10). Individual scores are represented as different-shaped points and group medians as black bold horizontal lines. The *X*-axis represents school-age population mean, and the red dashed horizontal line above represents the suggested clinical cut-point (90th percentile).

**Figure 1:**
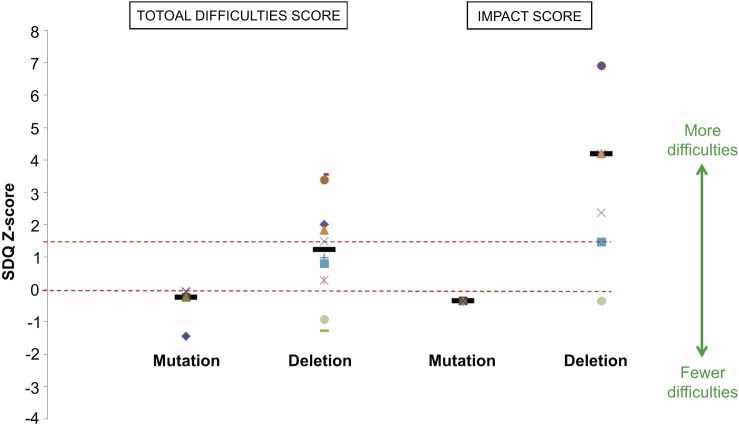
**Patient difficulties as shown by parent-reported Strengths and Difficulties Questionnaire (SDQ) scores (presented as *Z*-scores) for individuals <18 years with both *HNF1B* gene mutations (*n* = 4) and 17q12 microdeletions (*n* = 10).** Individual scores are represented as different-shaped points and group medians as black bold horizontal lines. The *X*-axis represents school-age population mean, and the red dashed horizontal line above represents the suggested clinical cut-point (90th percentile).

Use of the parent-reported Strengths and Difficulties Questionnaire (SDQ) revealed more patient difficulties in the deletion group with a median total difficulties score of 15.5 (IQR: 10, 20) compared with 7 in the mutation group (IQR: 3.5, 7.5; *P* = 0.048) (Figure 1). This is also higher than the mean total difficulties score of 8 ± 5.8 (SD) obtained in a normative sample of 10,438 British school-aged children.^33^ When analyzing the 4 subsections of the total difficulties score, conduct problems and peer relationship problems were more common in the deletion group as follows: median scores were 2.5 (IQR: 2, 5) and 4.5 (IQR: 1, 6), respectively, versus 0.5 (IQR: 0, 1) and 0 (IQR: 0, 0.5) in the mutation group, *P* = 0.04 and 0.02, respectively (Supplementary Figure S1). Five of the 10 children with a deletion scored above the suggested clinical cut-point of 15; all of these children apart from 1 had already been referred for further psychological evaluation.

Parental scores for the impact of these difficulties on the child’s life were similarly high in the deletion group with a median score of 5 (IQR: 2, 8). This was compared with a median score of 0 in the mutation group (IQR: 0, 0; *P* = 0.02) and a mean score of 0.4 ± 1.1 in the large normative sample mentioned previously.^33^

### Clinical diagnosis of neurodevelopmental disease in patients with a deletion

**Figure 2:**
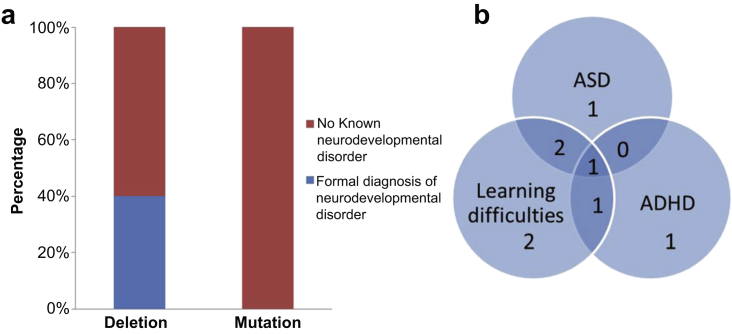
**Clinical diagnosis of neurodevelopmental disease.** (**a**) Stacked bar chart showing percentage of patients within both 17q12 microdeletion (*n* = 20) and *HNF1B* mutation (*n* = 18) groups with a known neurodevelopmental disorder including autism spectrum disorder (ASD), attention deficit hyperactivity disorder (ADHD), and/or learning difficulties requiring a Statement of Special Educational Needs or attendance at a special school. (**b**) Venn diagram illustrating the breakdown and overlap of diagnoses in the 8 patients with a deletion and neurodevelopmental disorder.

Eight of 20 participants (40%) with a deletion had a clinical diagnosis of either an ASD, attention deficit hyperactivity disorder (ADHD), and/or learning difficulties requiring a Statement of Special Educational Needs or current attendance at a special school compared with 0 of 18 with a mutation, *P* = 0.004 (Figure 2a). Of these 8 patients, 4 had comorbidity with learning difficulties accompanying a diagnosis of ASD and/or ADHD (Figure 2b, Supplementary Table S3). According to the second national survey of children’s mental health and well-being carried out in 2004, the prevalence of ASD in British children was 0.9% and hyperkinetic disorder/ADHD was 1.5%.^34^ Therefore, the frequency of ASD and ADHD found in participants with a deletion in this study far exceeds the baseline population rates.

### 17q12 deletions are associated with more autistic traits

**Figure 3:**
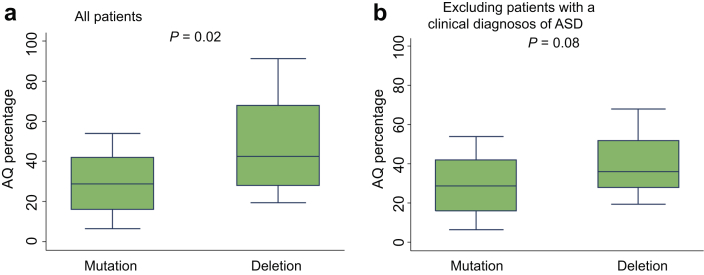
**Quantification of autistic traits using the Autism Spectrum Quotient (AQ) in individuals with HNF1B-associated disease of normal intelligence (defined as Intelligence quotient [IQ] > 69).** (**a**) Inclusion of all study patients with IQ > 69 (*n* = 36). (**b**) Exclusion of patients with a clinical diagnosis of an autism spectrum disorder ([ASD]; *n* = 33).

Patients with a deletion had a higher median Autism Spectrum Quotient (AQ) (43% [IQR: 28, 68] vs. 29% [IQR: 16, 42] in the mutation group, *P* = 0.02), indicating a greater number of autistic traits (Figure 3a). Although the AQ is not a diagnostic tool, cutoffs have been described for identifying individuals who may have clinically significant levels of autistic traits. However, referral for a full diagnostic assessment is only warranted if the individual is also suffering a degree of distress as a result of these traits.^35^, ^36^, ^37^ Six of 38 participants (16%) scored above the suggested cutoff; of these, all had a deletion and 3 of 6 had a confirmed diagnosis of ASD. To see whether the AQ results were being skewed by a small number of individuals with a high number of autistic traits, the analysis was repeated after excluding those with a known ASD. Although there was a trend toward a higher AQ in the deletion group (median AQ: 36% [IQR: 28, 52] vs. 29% [IQR: 16, 42] in the mutation group), this did not reach statistical significance (*P*= 0.08) (Figure 3b) but may have done so in a larger sample (*n* = 64).

### Cognitive ability is similar in both *HNF1B* mutation and deletion groups

**Figure 4:**
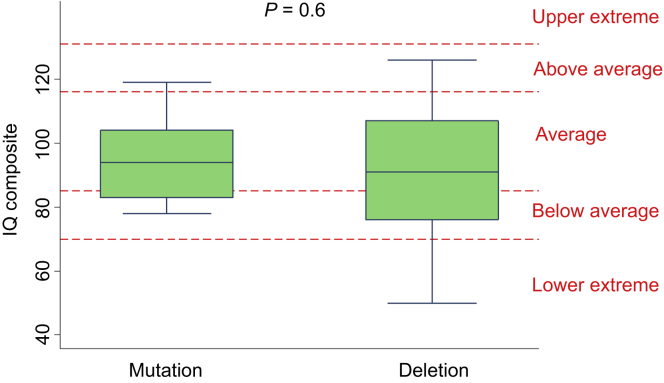
**Intelligence quotient (IQ) composite scores in individuals with HNF1B-associated disease**. Different IQ classifications are shown by the red dashed horizontal lines.

The median IQ composite was similar in both mutation and deletion groups (97 [IQR: 83, 104] vs. 91 [IQR: 76, 107]; *P* = 0.6) (Figure 4). Two participants with a deletion scored in the lower extreme category with IQ < 70.

### Facial dysmorphic features considered as a whole may be predictive of the presence of a 17q12 deletion

**Figure 5:**
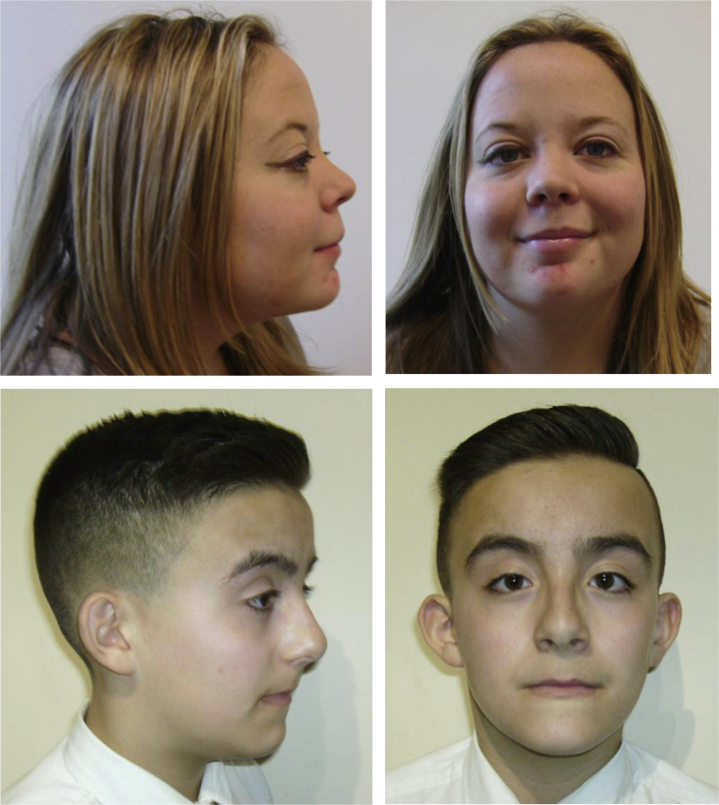
**Photographs of 2 study patients with a known *HNF1B* whole-gene deletion demonstrating the high forehead, high arched eyebrows, long philtrum, long face, and anteverted nares that, taken as a whole, suggest the presence of a deletion**.

Facial photographs were analyzed in 33 participants (18 with an intragenic *HNF1B* mutation, 15 with a deletion). None of the facial dysmorphic features previously described in association with the 17q12 deletion differed in frequency between the mutation and deletion groups (Supplementary Table S4). Variation in results between the 2 assessors was seen although overall interrater agreement was fair with a kappa coefficient of 0.4 (95% confidence interval: 0.3–0.5). When facial dysmorphic features were considered as a whole by both assessors to predict whether an individual had a deletion, sensitivity was 83% and specificity was 79% (Figure 5). Nine of 37 patients (24%) had a head circumference >90th percentile, but there was no difference in macrocephaly between the 2 groups (5 of 19 [26%] in deletion group vs. 4 of 18 [22%] in mutation group, *P* = 1).

## Discussion

The results of this study demonstrate that a neurodevelopmental phenotype is only seen in individuals with a 17q12 deletion. Compared with patients with an intragenic mutation, patients with a deletion had a greater number of autistic traits using the AQ and children displayed higher levels of psychopathology and impact on brief behavioral screening using the parent-reported SDQ. Indeed, 40% of participants with a deletion had been clinically diagnosed with a neurodevelopmental disorder; ASD and ADHD were seen much more commonly in the deletion group than were predicted from population prevalence rates. Most (17 of 18) of the patients with intragenic mutations had a nonsense or insertion/deletion loss of function mutation, predicted to result in reduced protein expression. The discrepancy in neurodevelopmental phenotype between the intragenic mutation and deletion groups suggests it is not simply haploinsufficiency of the *HNF1B* gene that is responsible for this aspect of the phenotype in individuals with a 17q12 deletion.

Our findings highlight the importance for nephrologists to be aware of this association between 17q12 microdeletion and neurodevelopmental disease to ensure referral to psychiatric services where appropriate. The features of conditions such as ASD can range from mild to severe and can also fluctuate over time and in response to different life events; this variable expression adds to the diagnostic challenges posed by these disorders.^38^ Individuals with a deletion and their families should be informed of the increased risk of a neurodevelopmental disorder so they can report any concerning symptoms if they arise to allow prompt investigation.

The results of this study contrast with recent work concluding that when children are diagnosed with a 17q12 deletion secondary to renal abnormalities, the neurodevelopmental phenotype is less severe than previously suggested in the literature.^20^ In this French cohort, only 1 of 26 patients with an *HNF1B* whole-gene deletion were diagnosed with autism as compared to 0 of 13 in the mutation group. However, the percentage of children with normal school progression requiring no educational support was lower in the deletion group (62.5% vs. 82%). It is possible that the lack of statistical difference between the 2 groups in terms of psychomotor development, school progression, and educational support may be explained by the younger age at study inclusion. Although both studies included a similar number of participants and all had HNF1B-associated disease identified secondary to renal disease or diabetes, the median age at inclusion was only 5.5 years (range 0.8–17) compared with 17 years (range 4–65) in our UK cohort. Schooling difficulties cannot be assessed in the very young and the features of neurodevelopmental disease may be more apparent as children become older; the median age at diagnosis of ASD, ADHD, and learning difficulties in our cohort was 8 years (IQR: 5.5, 9.5). Earlier work from another French cohort of 53 children with hyperechogenic or cystic kidneys and a 17q12 deletion reported autism in 3 cases (5.7%), a greater proportion than predicted from the prevalence of ASD in the pediatric population.^39^ This is in keeping with the increased frequency of ASD in deletion patients we described in our study.

When considered in isolation, none of the facial dysmorphic features previously described in association with a 17q12 deletion was statistically more common in the deletion group in this study. This contrasts with findings by Laffargue *et al.*,^20^ who reported that a high forehead, deep set eyes, and chubby cheeks were more frequently seen in the presence of a deletion rather than a mutation.^20^ However, when the craniofacial characteristics in our series were assessed as a whole, 2 independent clinical geneticists were able to predict the presence of a deletion with a sensitivity of 83% and specificity of 79%. This supports the prior hypothesis that the 17q12 deletion is associated with a mild but characteristic facial phenotype^22^ and that another genetic mechanism besides *HNF1B* haploinsufficiency is causative.

Interestingly, we found that patients with an intragenic *HNF1B* mutation had a significantly lower median estimated GFR than patients with a deletion, although this is unlikely to be related to the neurodevelopmental differences between the 2 groups. Ulinski *et al.*^7^ described the phenotype of 25 children with HNF1B-associated renal disease and found no difference in renal function between individuals with an *HNF1B* whole-gene deletion and those with point mutations. A later series that included 75 patients with HNF1B-associated renal disease showed that the proportion of individuals with renal impairment was significantly higher in those with a truncating mutation (nonsense, frameshift, or splice site) than in those with a deletion (*P* = 0.01).^5^ The investigators hypothesized that the older age of the patients with truncating mutations may partly explain the difference in renal function between the 2 groups; however, the mutation and deletion groups in our study were similarly matched in terms of median age. Seventeen of 18 intragenic mutations described in our series were truncating.

The results from this study provide the first detailed description of the neurodevelopmental phenotype of both children and adults diagnosed with HNF1B-associated disease. Both mutation and deletion groups were similarly matched in terms of general characteristics, and participants were systematically assessed for neurodevelopmental features using validated screening tools. However, several limitations were associated with this work. Despite inviting all eligible patients from the 4 different sites to take part, the study cohort represented only 45% of the total due to either inability to contact individuals despite several attempts or a negative response to participation. Therefore, the exact prevalence and spectrum of neurodevelopmental disorders in HNF1B-associated renal disease and diabetes remains unknown. Although individuals were systematically assessed using a combination of screening tools, participant/parent interview, and review of medical records, comprehensive screening tools and diagnostic tests for ASD and ADHD were not used. This means less severe disease may have been missed. Finally, genetic screening for other known causes of neurodevelopmental disease (e.g., Fragile X, other copy number variants) was not undertaken.

None of the patients with an intragenic *HNF1B* mutation in our study had a diagnosis of ASD, ADHD, or significant learning difficulties. Five individuals with HNF1B-associated disease secondary to gene mutation and either learning difficulties and/or epilepsy have been described, although other genetic causes were not excluded.^6^, ^12^, ^29^ To date, there have been no reports of *HNF1B* intragenic mutation and either ASD or ADHD presented in the literature. This supports our hypothesis that it is not haploinsufficiency of the *HNF1B* gene that is responsible for this aspect of the phenotype in individuals with a 17q12 deletion. It also highlights that further work is needed in this area to determine the cause of the phenotypic variability seen in these patients.

In summary, 17q12 microdeletions but not intragenic mutations are associated with a neurodevelopmental phenotype. All affected families should be informed of this risk and referred for appropriate psychiatric assessment if concerning symptoms arise.

## Materials and Methods

### Recruitment and *HNF1B* genetic analysis

Participants were recruited from January 31, 2013 to October 10, 2015 from 4 sites in the United Kingdom (adult renal and diabetes units at the Royal Devon and Exeter Hospital; pediatric renal units at Great Ormond Street Hospital for Children, Evelina London Children’s Hospital, and Birmingham Children’s Hospital). Inclusion criteria included the presence of either an *HNF1B* intragenic mutation or whole-gene deletion on genetic testing performed due to underlying renal abnormalities or diabetes and current age ≥4 years. All eligible patients were invited to participate. Informed written consent was obtained from all adult participants and parents of child participants, with assent from those aged <16 years. The study was conducted in agreement with the Declaration of Helsinki principles and approved by a regional ethics committee (National Research Ethics Service Committee South West—Frenchay). A total of 38 patients from 28 unrelated families with HNF1B-associated disease agreed to participate.

Initial mutation screening was performed by sequencing of coding exons and exon-intron boundaries together with gene dosage assessment by multiplex ligation-dependent probe amplification as previously described.^8^, ^19^ Droplet digital PCR was used to confirm the presence of an approximate 1.3 Mb deletion at chromosome 17q12 in the 20 patients with an *HNF1B* whole-gene deletion. This assay measured gene dosage for *ZNHIT3* and *HNF1B*, the most 5′ and 3′ genes within the recurrent 1.3 Mb 17q12 deletion. Droplet digital PCR was performed using the Bio-Rad QX200 (Bio-Rad Laboratories, Hercules, CA) and following standard protocols. Briefly, a reaction mix containing 22 ng genomic DNA, primers, and QX200 ddPCR EvaGreen supermix (Bio-Rad Laboratories) was subjected to the automated QX200 Droplet Generator (Bio-Rad Laboratories) to produce emulsions according to the manufacturer’s instructions. After PCR using a standard thermocycler (Bio-Rad Laboratories), sample fluorescence was assessed by the QX200 Droplet Reader (Bio-Rad Laboratories) and absolute quantification of amplified DNA product was determined by Poisson distribution using QuantaSoft software (Bio-Rad Laboratories). A full methodology, including primer sequences, is available on request.

### Clinical evaluation

Renal and extra-renal involvement in participants, including neurodevelopmental disorders, was documented using a standardized assessment of medical records and participant/parent interview in all cases plus educational psychology reports where available. An Index of Multiple Deprivation 2007 score was derived for each participant using their postcode and was used as an overall measure of deprivation. Imaging results from ultrasonography, computed tomography, or magnetic resonance imaging were reviewed to look for kidney, pancreas, and genital tract abnormalities. GFR was estimated using the (i) Schwartz-Haycock formula in children,^40^ optimized for children with renal malformations assessed in each individual pediatric renal unit where possible, and (ii) simplified Modification of Diet in Renal Disease formula in adults.^41^ GFR was set at 0 for patients on renal replacement therapy. Proteinuria was defined as albumin-creatinine ratio >30 mg/mmol or protein-creatinine ratio >50 mg/mmol. Hypomagnesemia was defined as serum magnesium <0.7 mmol/l and hyperuricemia as a serum urate level above the upper limit of the normal reference range for age and sex from the analyzing laboratory. Diabetes was diagnosed either according to World Health Organization guidelines or on the basis of established treatment with oral hypoglycemic agents/insulin. Abnormal liver function tests were defined as serum alanine aminotransferase, aspartate transaminase, gamma-glutamyl transferase, or alkaline phosphatase levels above the upper limit of the normal reference range for age and sex from the analyzing laboratory.

Brief behavioral screening was carried out in 4- to 16-year-olds using the SDQ.^42^ The questionnaire was completed by parents and included 25 items on psychological attributes covering 5 areas: (i) emotional symptoms, (ii) conduct problems, (iii) hyperactivity/inattention, (iv) peer relationship problems, and (v) prosocial behavior. Scores from areas 1 to 4 were added together to generate a total difficulties score. An impact supplement was also administered, which provided further information on chronicity, distress, social impairment, and burden to others if the child was felt to have a problem.

Autistic traits were assessed using the AQ in participants of normal intelligence (defined in this study as IQ ≥ 70). Three different versions of this questionnaire were available from the Autism Research Centre depending on participant age: child (completed by the parent of each child participant aged 4–11 years), adolescent (completed by the parent of each child participant aged 12–15 years), and adult (completed by each participant aged ≥16 years).^35^, ^36^, ^37^ AQ scores were converted to percentages for standardization between the different age groups. Cognitive ability was assessed in all participants using the Kaufman Brief Intelligence Test.^43^ This is an individually administered measure of verbal and nonverbal intelligence, which yields an overall score known as the IQ composite (an age-based standard score with a mean ± SD of 100 ± 15).

Facial photographs of participants were taken and assessed by 2 experienced clinical geneticists for dysmorphic features previously associated with the 17q12 deletion. The assessors were blinded to the genetic status of each participant. Head circumference was measured and converted to a percentile using British 1990 (UK90) growth reference charts for children and separate centile charts for adults.^44^ Macrocephaly was defined as head circumference >90th percentile.

### Statistical analysis

Qualitative variables were described with percentages and quantitative variables with median and IQR. Differences between *HNF1B* gene mutation and deletion groups were assessed using the Fisher exact test for categorical variables and the Mann-Whitney *U* test for continuous variables. A *P*-value of <0.05 was considered to be statistically significant. The Bonferroni method was used to correct for multiple comparisons when evaluating dysmorphic features and interrater agreement between the 2 independent assessors was quantified using the Cohen kappa coefficient. All analyses were carried out using StataSE (version 13.1, StataCorp, College Station, TX) and GraphPad statistical software (GraphPad Software, La Jolla, CA).

## Disclosure

All the authors declared no competing interests.

## Figures and Tables

**Table 1:** Characteristics of study patients with either an *HNF1B* intragenic mutation or 17q12 microdeletion

	*HNF1B* mutation (*n* = 18)	17q12 microdeletion (*n* = 20)	*P*
A**ge, yr**	19 (13, 45)	15.5 (11, 35)	0.3
**Sex**	M 8 (44%), F 10 (56%)	M 8 (40%), F 12 (60%)	1
**Ethnicity**	White 18 (100%)	White 19 (95%), mixed 1 (5%)	1
**Indices of Deprivation 2007 score**	25 (16, 46)	21 (12, 30)	0.4
**Renal phenotype**			
**Renal abnormality**			
**Cysts/cystic dysplasia**	12 (67%)	17 (85%)	0.3
**Other**^a^	4 (22%)	3 (15%)	
**Unknown**	2 (11%)	0	
A**ge at diagnosis of renal disease, yr**	0 (0, 20)	0 (0, 24)	0.7
**Renal replacement therapy**	3 (17%)	1 (5%)	0.3
**GFR, m**l**/min/1.73 m**^**2**^	42.6 (31, 60)	81.4 (56, 91)	**0.002**
**Proteinuria**^b^^,^^c^	2 (13%)	2 (11%)	1
**Serum magnesium,**^c^**mmol/l**	0.7 (0.67, 0.75)	0.58 (0.53, 0.69)	**0.01**
**Hypomagnesemia**^c^^,^^d^	6 (40%)	12 (63%)	0.3
**Hyperuricemia**^c^^,^^e^	10 (67%)	3 (16%)	**0.004**
**Gout**	6 (33%)	2 (10%)	0.1
**Extra-renal phenotype**			
**Pancreas**			
**Diabetes**	7 (39%)	8 (40%)	1
**Age at diagnosis of diabetes**	19 (18, 37)	29 (17, 32)	1
**Pancreatic hypoplasia**^f^^,^^g^	1 (6%)	5 (25%)	0.2
**Fecal elastase, μg/g stool**	402.5 (170, 500)	280 (167, 433)	0.8
**Genital tract**			
**Genital tract malformation**^g^^,^^h^	1 (6%)	2 (10%)	1
**Liver**			
**Abnormal liver function tests**^g^	5 (28%)	6 (30%)	1

Values are median (IQR) or *n* (%). Bold *P*-values are statistically significant.

F, female; GFR, glomerular filtration rate; IQR, interquartile range; M, male.

a Other renal structural abnormalities included single kidney, collecting system abnormalities, and bilateral hydronephrosis.

b Proteinuria defined as albumin-creatinine ratio >30 mg/mmol or protein-creatinine ratio >50 mg/mmol.

c Only assessed in individuals with native renal function.

d Hypomagnesemia defined as serum magnesium <0.7 mmol/l.

e Hyperuricemia defined as serum urate level above upper limit of normal reference range for age and sex from analyzing laboratory.

f Hypoplasia of body and/or tail of pancreas.

g Not systematically assessed for.

h Genital tract malformations included (i) unilateral undescended testicle and blind-ending epididymis, (ii) bilateral undescended testicles, and (iii) bicornuate uterus.

## Supplementary Material

Table S1 Details of intragenic *HNF1B* mutations.

Table S2 General characteristics of participants and nonparticipants who were eligible to take part in the study. F, female; IQR, interquartile range; M, male. ^†^All had either an *HNF1B* point mutation or whole-gene deletion on previous genetic testing and current age ≥4 years. *Other renal structural abnormalities included single kidney, collecting system abnormalities and bilateral hydronephrosis; in 5 cases the imaging results were not known.

Table S3 Details of study patients with HNF1B-associated disease and a clinically diagnosed neurodevelopmental disorder. ADHD, attention deficit hyperactivity disorder; ASD, autism spectrum disorder; AQ, Autism Spectrum Quotient; IQ, intelligence quotient; SDQ, Strengths and Difficulties Questionnaire.

Table S4 Assessment of facial dysmorphic features in study patients with either *HNF1B* mutation or 17q12 microdeletion by 2 independent clinical geneticists (Charles Shaw-Smith and Peter Turnpenny). ^†^The presence of a clinical feature was determined using the results of 1 or both assessors; interrater agreement between the 2 assessors was quantified using the Cohen kappa coefficient. *The Bonferroni method was used to correct for multiple comparisons; statistical significance was reset at *P* = 0.004 (0.05/13).
