# Supplementary material for: Chromosome 17q12 microdeletions but not intragenic HNF1B mutations link developmental kidney disease and psychiatric disorder
Source: Kidney Int. 2016 Jul;90(1):203–11. doi: 10.1016/j.kint.2016.03.027 (PMC4915913; doi:10.1016/j.kint.2016.03.027)
Supplement: Table S1 — Details of intragenic HNF1B mutations. [file mmc1.docx]

**Supplementary Table 1** Details of intragenic *HNF1B* mutations

| Patient study number | Nucleotide change | Amino acid change | Functional effect | Inheritance |
| --- | --- | --- | --- | --- |
| 2 | c.982_986delCCTCT | p.P328fs | Frameshift | *De novo* |
| 4 | c.982_986delCCTCT | p.P328fs | Frameshift | Maternal (son of patient 2) |
| 6 | c.1138delG | p.V380fs | Frameshift | *De novo* |
| 10 | c.544+3_544+4insT | p.? | Splice site | Maternal (daughter of patient 13) |
| 11 | c.544+3_544+4insT | p.? | Splice site | Maternal (daughter of patient 10) |
| 12 | c.544+3_544+4insT | p.? | Splice site | Maternal (son of patient 10) |
| 13 | c.544+3_544+4insT | p.? | Splice site | Unknown |
| 14 | c.544+3_544+4insT | p.? | Splice site | Unknown (half-sister of patient 13) |
| 20 | c.1235dupC | p.V413fs | Frameshift | Maternal (son of patient 23) |
| 21 | c.1006dup | p.H336fs | Frameshift | Unknown |
| 22 | c.1006dup | p.H336fs | Frameshift | Paternal (son of patient 21) |
| 23 | c.1235dupC | p.V413fs | Frameshift | Unknown |
| 24 | c.541C>T | p.R181* | Nonsense | Paternal |
| 25 | c.541C>T | p.R181* | Nonsense | Paternal |
| 26 | c.541C>T | p.R181* | Nonsense | Paternal |
| 31 | c.398A>G | p.N133S | Missense | *De novo* |
| 38 | c.544C>T | p.Q182* | Nonsense | *De novo* |
| 39 | c.1048dup | p.V350fs | Frameshift | Unknown |
