# Supplementary material for: Chromosome 17q12 microdeletions but not intragenic HNF1B mutations link developmental kidney disease and psychiatric disorder
Source: Kidney Int. 2016 Jul;90(1):203–11. doi: 10.1016/j.kint.2016.03.027 (PMC4915913; doi:10.1016/j.kint.2016.03.027)
Supplement: Table S2 — General characteristics of participants and nonparticipants who were eligible to take part in the study. F, female; IQR, interquartile range; M, male. †All had either an HNF1B point mutation or whole-gene deletion on previous genetic testing and current age ≥4 years. *Other renal structural abnormalities included single kidney, collecting system abnormalities and bilateral hydronephrosis; in 5 cases the imaging results were not known. [file mmc2.docx]

**Supplementary Table 2** General characteristics of participants and non-participants who were eligible to take part in the study

|  | Participants  *N*=38 | Non-participants^ᵻ^  *N*=47 | *P* |
| --- | --- | --- | --- |
| Genetic abnormality, *N* (%)  Mutation  Whole-gene deletion | 18 (47)  20 (53) | 17 (36)  30 (64) | *0.4* |
| Median age, years (IQR) | 17 (12-38) | 14 (11-20) | *0.06* |
| Sex, *N* (%) | M 16 (42), F 22 (58) | M 24 (51), F 23 (49) | *0.5* |
| Ethnicity, *N* (%)  White  Mixed  Asian  Black/African/Caribbean  Unknown | 37 (97)  1 (3) | 20 (43)  2 (4)  9 (19)  3 (6)  13 (28) | ***<0.001*** |
| Median Indices of Deprivation 2007 score (IQR) | 23 (13-36) | 25 (15-36) | *0.9* |
| Renal abnormality, *N* (%)  Cysts/cystic dysplasia  Other* | 29 (81)  7 (19) | 37 (82)  8 (19) | *1* |
| Abbreviations: F, female; IQR, interquartile range; M, male.  ^ᵻ^All had either an *HNF1B* point mutation or whole-gene deletion on previous genetic testing and current age ≥4 years.  *Other renal structural abnormalities included single kidney, collecting system abnormalities and bilateral hydronephrosis; in 5 cases the imaging results were not known. | | | |
