# Supplementary material for: Chromosome 17q12 microdeletions but not intragenic HNF1B mutations link developmental kidney disease and psychiatric disorder
Source: Kidney Int. 2016 Jul;90(1):203–11. doi: 10.1016/j.kint.2016.03.027 (PMC4915913; doi:10.1016/j.kint.2016.03.027)
Supplement: Table S3 — Details of study patients with HNF1B-associated disease and a clinically diagnosed neurodevelopmental disorder. ADHD, attention deficit hyperactivity disorder; ASD, autism spectrum disorder; AQ, Autism Spectrum Quotient; IQ, intelligence quotient; SDQ, Strengths and Difficulties Questionnaire. [file mmc3.docx]

**Supplementary Table 3** Details of study patients with HNF1B-associated disease and a clinically diagnosed neurodevelopmental disorder

| Patient study number | Age (years) | Sex | *HNF1B* genetic abnormality | Details of neurodevelopmental disorder | SDQ scores | | AQ | IQ |
| --- | --- | --- | --- | --- | --- | --- | --- | --- |
|  |  |  |  |  | **Total difficulties score** | **Impact score** |  |  |
| 5 | 14 | Male | Whole-gene deletion  (c.1-?_1674+?del) | ADHD and dyspraxia diagnosed aged 5 years; treatment with methylphenidate; attendance at mainstream school with Statement of Special Educational Needs. |  |  | 68% | 114 |
| 8 | 33 | Male | Whole-gene deletion  (c.1-?_1674+?del) | Learning difficulties with attendance at special school; Asperger’s syndrome diagnosed in early twenties. |  |  | 63% | 69 |
| 9 | 8 | Female | Whole-gene deletion  (c.1-?_1674+?del) | ASD diagnosed aged 8 years. | 17 | 3 | 91% | 107 |
| 16 | 9 | Female | Whole-gene deletion  (c.1-?_1674+?del) | Difficulties with literacy and numeracy skills, attention and concentration plus speech and language skills; attendance at mainstream school with Statement of Special Educational Needs recommending 9.25 hours of additional specialist teaching support per week. | 10 | 5 | 41% | 76 |
| 27 | 12 | Male | Whole-gene deletion  (c.1-?_1674+?del) | Extreme delayed speech with no words, requiring speech and language input; ASD diagnosed aged 5 years with attendance at special school for children with autism since age 8 years. | 19 | 5 | 84% | 76 |
| 33 | 14 | Male | Whole-gene deletion  (c.1-?_1674+?del) | ASD and ADHD diagnosed aged 11 years after long history of challenging behaviours at home and school; attendance at special school for children with emotional and behavioural difficulties. | 29 | 8 |  |  |
| 34 | 16 | Female | Whole-gene deletion  (c.1-?_1674+?del) | Specific learning difficulties (dyslexic), coordination difficulties (dyspraxia) plus receptive and expressive language delay diagnosed aged 8 years; attendance at mainstream school with Statement of Special Educational Needs recommending 15 hours of additional specialist teaching support per week. | 14 | 5 | 34% | 50 |
| 35 | 13 | Male | Whole-gene deletion  (c.1-?1674+?del) | Attention Deficit Disorder diagnosed aged 6 years; treatment with methylphenidate. | 28 | 8 | 28% | 96 |
| Abbreviations: ADHD, attention deficit hyperactivity disorder; ASD, autism spectrum disorder; AQ, Autism Spectrum Quotient; IQ, intelligence quotient; SDQ, Strengths and Difficulties Questionnaire. | | | | | | | | |
