# Supplementary material for: Chromosome 17q12 microdeletions but not intragenic HNF1B mutations link developmental kidney disease and psychiatric disorder
Source: Kidney Int. 2016 Jul;90(1):203–11. doi: 10.1016/j.kint.2016.03.027 (PMC4915913; doi:10.1016/j.kint.2016.03.027)
Supplement: Table S4 — Assessment of facial dysmorphic features in study patients with either HNF1B mutation or 17q12 microdeletion by 2 independent clinical geneticists (Charles Shaw-Smith and Peter Turnpenny). †The presence of a clinical feature was determined using the results of 1 or both assessors; interrater agreement between the 2 assessors was quantified using the Cohen kappa coefficient. *The Bonferroni method was used to correct for multiple comparisons; statistical significance was reset at P = 0.004 (0.05/13). [file mmc4.docx]

**Supplementary Table 4** Assessment of facial dysmorphic features in study patients with either *HNF1B* mutation or 17q12 microdeletion by two independent clinical geneticists (Charles Shaw-Smith and Peter Turnpenny)

| Feature^ᵻ^ | *HNF1B* mutation  (n=18) | 17q12 microdeletion (n=15) | *P** | Inter-rater agreement | |
| --- | --- | --- | --- | --- | --- |
|  |  |  |  | **Cohen's kappa coefficient**  **(95% confidence interval)** | **Strength of agreement** |
| High forehead | 10 (56%) | 13 (87%) | 0.07 | 0.2 | Fair |
| Arched & high eyebrows | 8 (44%) | 8 (53%) | 0.7 | 0.6 | Moderate |
| Epicanthal folds | 0 | 0 | 1 | - | - |
| Downslanting palpebral fissures | 2 (11%) | 4 (27%) | 0.4 | 0.6 | Good |
| Deep set eyes | 4 (22%) | 3 (20%) | 1 | -0.1 | Worse than expected by chance alone |
| Ptosis | 5 (28%) | 7 (47%) | 0.3 | 0.4 | Fair |
| Depressed nasal bridge | 0 | 3 (20%) | 0.08 | -0.04 | Worse than expected by chance alone |
| Long philtrum | 5 (28%) | 9 (60%) | 0.09 | 0.2 | Poor |
| Malar flattening | 6 (33%) | 10 (67%) | 0.08 | 0.4 | Fair |
| Full cheeks | 4 (22%) | 5 (33%) | 0.7 | 0.4 | Moderate |
| Long face | 12 (67%) | 12 (80%) | 0.5 | 0.3 | Fair |
| Facial asymmetry | 1 (6%) | 3 (20%) | 0.3 | -0.07 | Worse than expected by chance alone |
| Anteverted nares | 6 (33%) | 13 (87%) | 0.004 | 0.2 | Fair |
| ^ᵻ^The presence of a clinical feature was determined using the results of one or both assessors; inter-rater agreement between the two assessors was quantified using Cohen's kappa coefficient  *The Bonferroni method was used to correct for multiple comparisons; statistical significance was re-set at *P* <0.05/13=0.004 | | | | | |
